# LncRNA LUCAT1/miR-181a-5p axis promotes proliferation and invasion of breast cancer via targeting KLF6 and KLF15

**DOI:** 10.1186/s12860-020-00310-0

**Published:** 2020-09-30

**Authors:** Yun Liu, Teng Cheng, Yaying Du, Xiaopeng Hu, Wenfei Xia

**Affiliations:** 1 Department of ENT, Tongji Hospital, Tongji Medical College, Huazhong University of Science and Technology, Wuhan City, Hubei Province 430030 PR China; 2 Department of Breast and Thyroid surgery, Division of General Surgery, Tongji Hospital, Tongji Medical College, Huazhong University of Science and Technology, No. 1095, Jiefang Avenue, Qiaokou District, Wuhan City, Hubei Province 430030 PR China

**Keywords:** LUCAT1, miR-181a-5p, Breast cancer, KLF6, KLF15

## Abstract

**Background:**

Long non-coding RNAs (lncRNAs) are novel regulatory molecules in breast cancer development. LncRNA LUCAT1 is a potential tumor promoter in human cancers. In this study, we aimed to explore the role of LUCAT1 in human breast cancer tissues and cells.

**Methods:**

A total of 31 breast cancer patients who underwent tumor resection, but without chemo- or radiotherapy or acute lung/heart/kidney diseases, provided tumor and adjacent normal tissues. Bioinformatic analysis, qRT-PCR, and luciferase reporter assay were carried out during the study.

**Results:**

qRT-PCR analysis indicated that, compared with the adjacent tissues and MCF-10A normal breast epithelial cells, LUCAT1 was markedly up-regulated in the breast cancer tissues and five BC cell lines, including MDA-MB-231, MDA-MB-468, MDA-MB-435, SKBR3, and MCF-7. The knockdown of LUCAT1, through the transfection of small interfering RNA (siRNA) specific to LUCAT1, resulted in inhibition of proliferation in breast cancer cells. The expression levels of miR-181a-5p were decreased in the breast cancer tissues and five BC cell lines. Bioinformatic analysis and luciferase reporter assay suggested the interaction between miR-181a-5p and LUCAT1. In addition, the effects of LUCAT1 on promoting cell proliferation were attenuated by overexpression of miR-181a-5p through the transfection of miR-181a-5p mimic. Moreover, bioinformatics and luciferase reporter assay confirmed that miR-181a-5p targeted the 3′-UTR region of KLF6 and KLF15 mRNA, which were two tumor suppressor genes. LUCAT1/miR-181a-5p axis regulated the expression of KLF6 and KLF15 both in vitro and in vivo.

**Conclusions:**

Our data indicate that LUCAT1/miR-181a-5p axis can serve as a novel therapeutic target in breast cancer.

## Background

Breast cancer (BC) is the second leading cause of cancer-related death in women [1]. While huge improvement of the survival of BC patients with localized cancer has been made, treatment of late-stage cancers, which are metastatic and characterized by chemoresistance, remains a clinical challenge [2]. There is a continuous search for new biomarkers of BC.

Emerging evidences has indicated that long non-coding RNAs (lncRNAs) and microRNAs (miRNAs), which are two classes of non-coding RNAs in human genome, are important regulators of cancers by serving as post-transcriptional regulators that activate or suppress oncogenes to mediate the proliferation, invasion, metastasis and chemoresistance of cancer cells [3, 4]. LncRNAs are of over 200 nucleotides in length and miRNAs are of 21–25 nucleotides in length. In breast cancer, a number of lncRNAs and miRNAs are found as potent regulators, which are utilized as both diagnostic and therapeutic targets, providing promising targets of novel molecular therapies in advancing the BC clinical management [4, 5].

LncRNA LUCAT1 is recently characterized as a tumor-promoting lncRNA in a wide variety of cancers, including osteosarcoma [6], non-small cell lung cancer [7], esophageal cancer [8], and renal cell carcinoma [9]. Importantly, LUCAT1 was shown to be associated with tumorigenesis [8] and poor prognosis of cancer patients [7] and participated in chemoresistance [7]. This study aimed to investigate the role of LUCAT1 in BC.

MiR-181a-5p is a miRNA shown to play an essential role in multiple cancers, such as hepatic cancer [10], non-small cell lung cancer [11, 12] and human diseases, such as osteoarthritis [13]. The interaction between lncRNA CCAT1 and miR-181a-5p has been demonstrated in endometrial carcinoma [14]. However, the interaction between miR-181a-5p and LUCAT1 has not been verified in BC and the underlying mechanism of their regulation has not been fully elucidated.

KLF6 and KLF15 are two genes of the class of Kruppel-like factors. In BC, KLF6 was shown to inhibit cancer growth by inactivating estrogen-receptor mediated pathways [15]. KLF15 is also a putative BC suppressor gene [16]. KLFs could modulate the expression of ERα. And they all could inhibit the expression of ERα. Moreover, KLF6 and KLF15 are also targets of miRNAs [17, 18].

The present study aimed to investigate the role of the LUCAT1/miR-181a-5p axis in BC and the mechanism of their regulation. Both clinical tissues and BC cell lines were used to explore the potential of LUCAT1 and miR-181a-5p as biomarkers of BC. Overexpression and knockdown of LUCAT1 were induced through the transfection of overexpression plasmid and small-interfering RNAs (siRNAs) specific to LUCAT1, to explore the role of LUCAT1 in regulating BC proliferation in vitro and in vivo. Bioinformatic analysis and luciferase reporter assay were utilized to investigate the binding of LUCAT1 and miR-181a-5p to their targets.

## Results

### LUCAT1 is upregulated in breast cancer

**Fig. 1:**
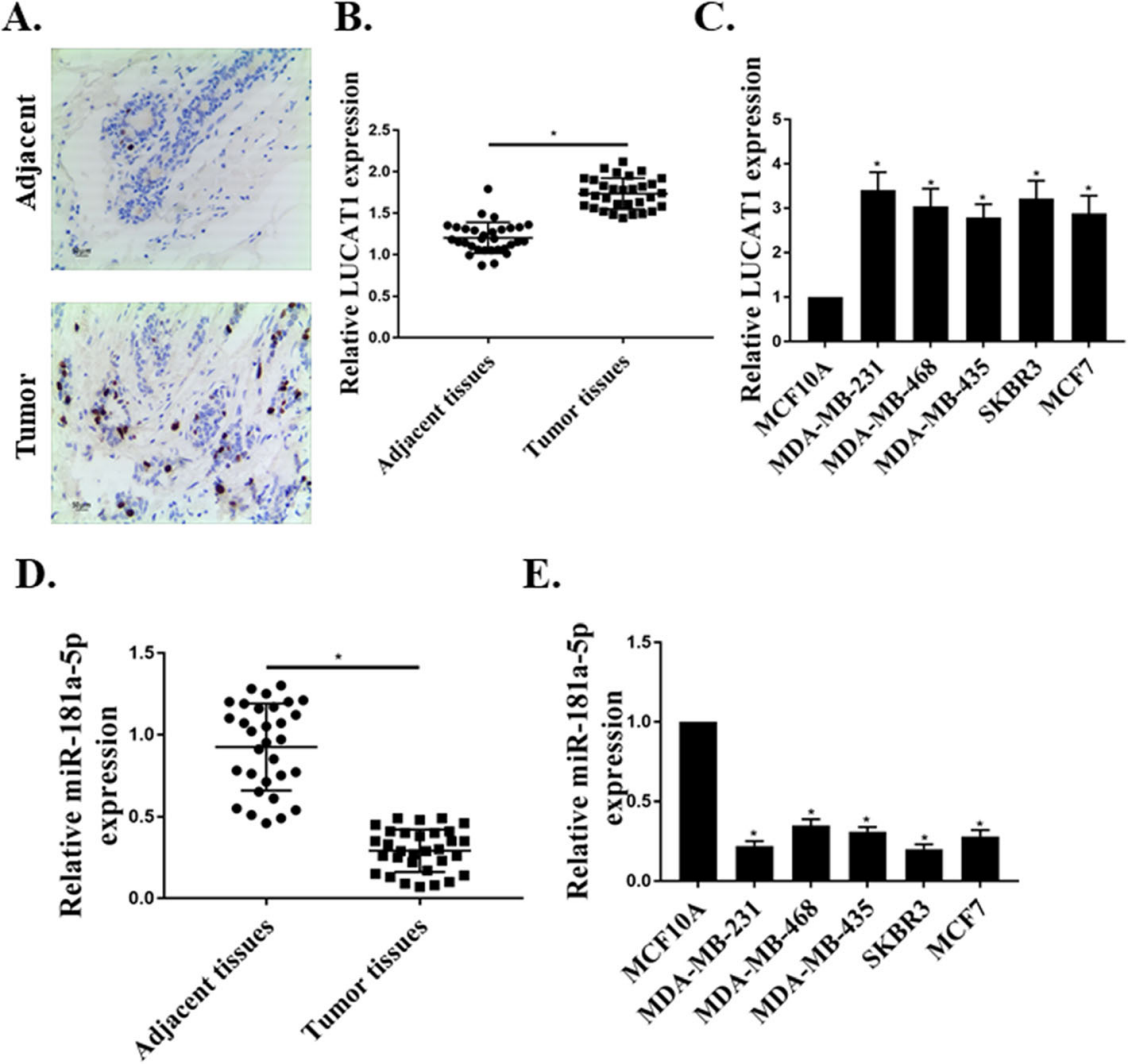
The expression of LUCAT1 and miR-181a-5p in tissues and cells. RT-qPCR was used to detect expression levels of LUCAT1 in BC tumor tissues and adjacent tissues (**a**, **b**), and cells including normal MCF-10A cells and breast cancer cell lines SKBR3, MCF7, MDA-MB-231, MDA-MB-468, MDA-MB-435 (**c**), demonstrating that LUCAT1 was overexpressed. RT-qPCR was used to detect expression levels of miR-181a-5p in BC tumor tissues and adjacent tissues (**d**), and cells including normal MCF-10A cells and breast cancer cell lines SKBR3, MCF7, MDA-MB-231, MDA-MB-468, MDA-MB-435, demonstrating that miR-181a-5p was expressed lowly (**e**). *p < 0.05, *n* = 31

The expression levels of LUCAT1 in BC tissues were compared with that of adjacent tissues, which showed a significant upregulation of LUCAT1 in BC tissues (*p* < 0.01, Fig. 1a and b). Further, the expression of LUCAT1 in different BC cells (MCF-7, MDA-MB-231, MDA-MB-468, MDA-MB-435, SKBR3) and normal breast cells (MCF-10A) were also evaluated. The results showed a considerable overexpression of LUCAT1 in the BC cell lines compared to that in MCF-10A cells (*p* < 0.05, Fig. 1c). Due to the prominent upregulation of LUCAT1 in MDA-MB-231 cells compared with that in other cell lines, MDA-MB-231 cells were used in the subsequent experiments. The MDA-MB-231 cell line used in our experiment was genotyped by STR genotype detection. The DNA typing of this cell line found a perfect match in the cell line search, and the ATCC database showed that the cell was named MDA-MB-231. No multiple alleles were found in this cell line (Table 1). In addition, miR-181a-5p was downregulated in BC tissues and BC cell lines (Fig. 1d and e).

**Table 1:** Genotyping of STR and Amelogenin loci in MDA-MB-231 cells

Marker	Allele 1	Allele 2
D5s818	12	12
D13S317	13	13
D7S820	8	9
D16S539	12	12
VWA	15	18
TH01	7	9.3
Amelogenin	X	X
TPOX	8	9
CSFIPO	12	13
FGA	22	23
Penta D	11	14
Penta E	11	11
D2S1338	20	21
D3S1358	16	16
D8S1179	13	13
D18S51	11	16
D19S433	11	14
D21S11	30	33.2

### LUCAT1 directly targets miR-181a-5p

**Fig. 2:**
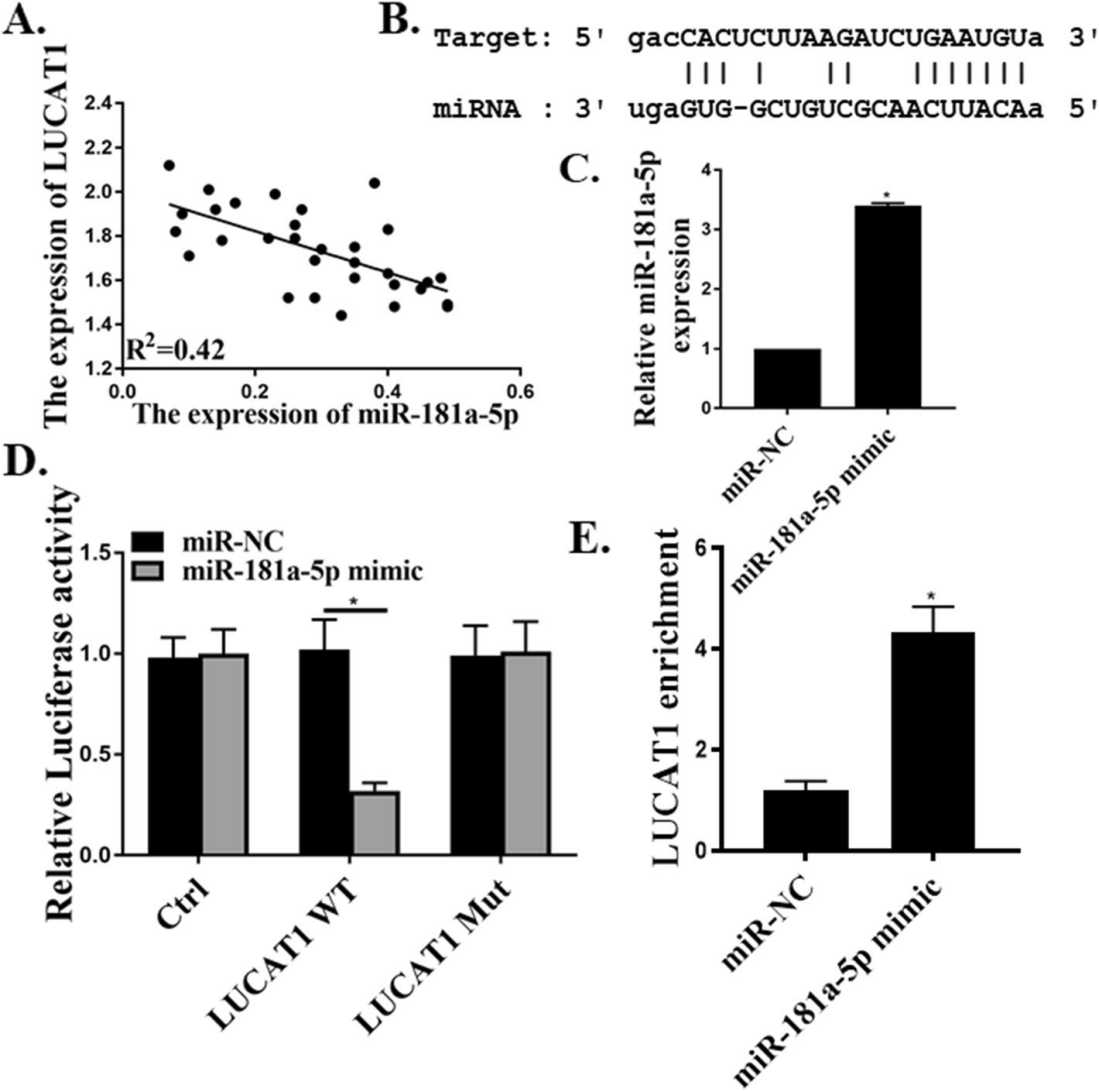
LUCAT1 directly targeted miR-181a-5p. **a** Pearson’s correlation analysis was used to determine the relationship between expression of miR-181a-5p and LUCAT1. **b** The targeting relation between lncRNA LUCAT1 and miR-181a-5p by database. **c** RT-qPCR examined the miR-181a-5p expression. **d** The dual luciferase assay. **e** The targeting relations of LUCAT1 and miR-181a-5p were confirmed by RNA pull-down assay. *p < 0.05, *n* = 5

A negative correlation between LUCAT1 and miR-181a-5p was found (Fig. 2a). Bioinformatics analysis (Starbase: http://starbase.sysu.edu.cn/) showed that LUCAT1 contained a binding site of miR-181a-5p (Fig. 2b). qRT-PCR assay confirmed the overexpression of miR-181a-5p in MDA-MB-231 cells after the transfection of miR-181a-5p mimic (Fig. 2c). The relationship between LUCAT1 and miR-181a-5p was then explored by luciferase reporter assay, which showed that overexpression of miR-181a-5p markedly suppressed the luciferase activity in the LUCAT1 WT group, but not in the LUCAT1 mutant group (Fig. 2d). To validate the direct interaction between miR-181a-5p and LUCAT1, biotinylated miR-181a-5p probe was tested to pull down LUCAT1 (*p* < 0.01, Fig. 2e). As a result, endogenous LUCAT1 was reduced specifically by miR-181a-5p probe, but not by the control probe, suggesting that miR-181a-5p directly bind to LUCAT1 to exert inhibitory effects.

### Downregulation of LUCAT1 reduces cell proliferation, migration, and invasion of breast cancer

**Fig. 3:**
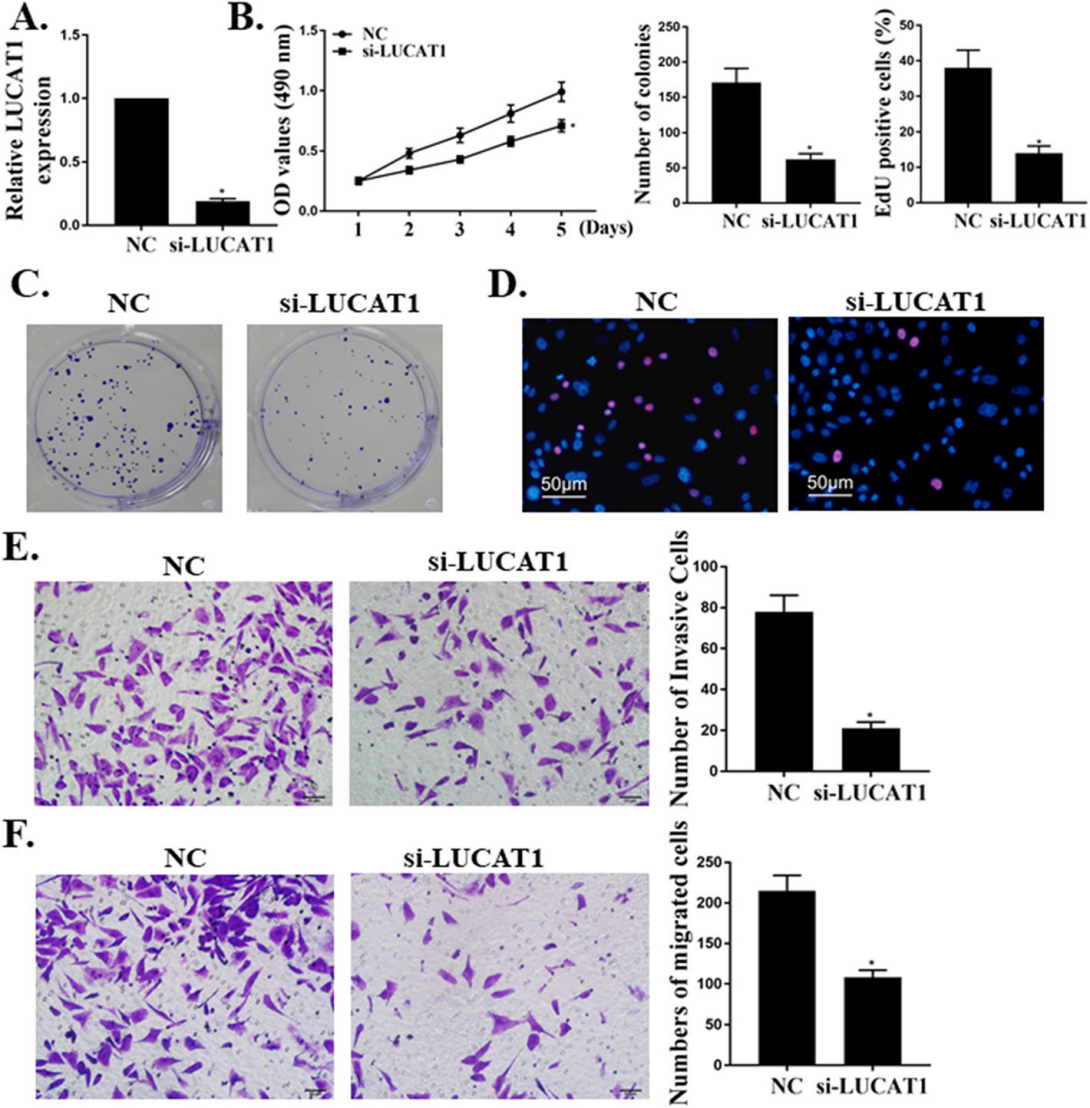
Downregulation of LUCAT1 reduces cell proliferation, migration and invasion in BC. **a** Transfection efficiency of si-LUCAT1 was confirmed by qRT-PCR method. **b** MTT assay: LUCAT1 knockdown in MDA-MB- 231 cells significantly inhibited cell proliferation. **c** Colony formation assay: colony formation of BC cells was decreased by knockdown of LUCAT1. **d** Transwell migration assay LUCAT1 knockdown in MDA-MB-231 cells reduced the migration of BC cells. **e** Transwell invasive assay: LUCAT1 knockdown significantly suppressed cell invasive capacity in BC cells. *p < 0.05

LUCAT1 was knocked-down and qRT-PCR results showed that the expression of LUCAT1 was downregulated (*p* < 0.01, Fig. 3a). MTT and colony formation assay revealed that knockdown of LUCAT1 reduced cell viability and proliferation, respectively (Fig. 3b and c). Moreover, knockdown of LUCAT1 significantly suppressed cell migratory and invasive capacities of MDA-MB-231 cells (Fig. 3d-f).

### MiR-181a-5p mediates the effects of LUCAT1 on cell proliferation, migration and invasion in breast cancer

**Fig. 4:**
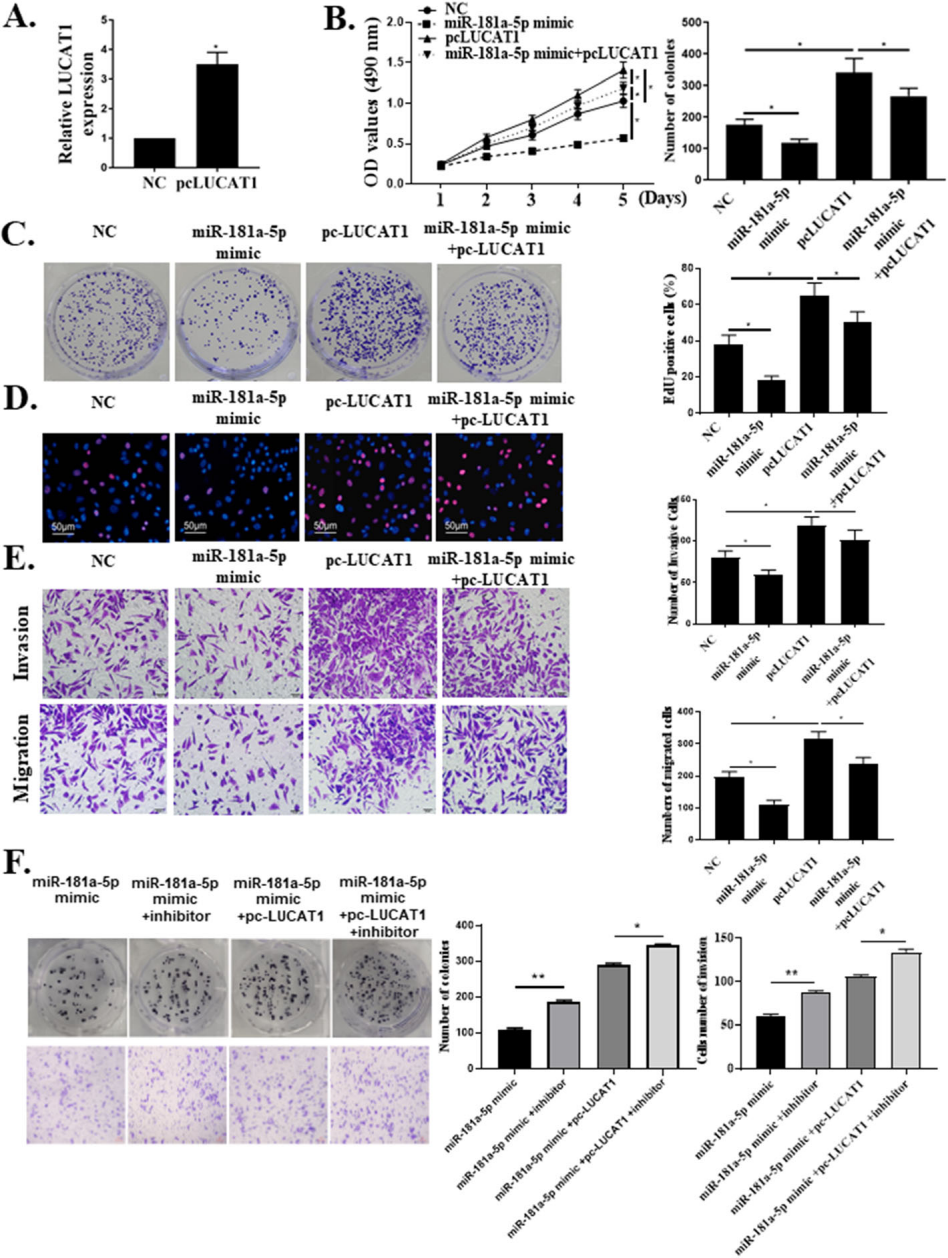
The effects of LUCAT1 on cell proliferation, migration and invasion were mediated by miR-181a-5p in BC. **a** The transfection efficiency of pc-LUCAT1 was confirmed by qRT-PCR method. **b**-**e** MTT assay, colony formation assay, transwell migration assay and transwell invasive assay: treated with NC, miR-181a-5p mimic, pc-LUCAT1, and miR-181a-5p mimic + pc-LUCAT1 respectively in MDA-MB-231 cells. **f** Colony formation assay and transwell invasive assay: treated with respectively miR-181a-5p mimic, miR-181a-5p mimic + inhibitor, miR-181a-5p mimic + pc-LUCAT1, and miR-181a-5p mimic + pc-LUCAT1 + inhibitor in MDA-MB-231 cells. *Compared with NC group, *p < 0.05, **p < 0.01

LUCAT1 was then overexpressed by transfecting an overexpression plasmid containing LUCAT1, named pc-LUCAT1 (*p* < 0.01, Fig. 4a). MTT and colony formation assay indicated that overexpression of miR-181a-5p (miR-181 mimic group) reduced cell viability compared with the miR-NC transfection (control group), while cells transfected with pc-LUCAT1 exhibited remarkably increased cell proliferation. Co-transfection of pc-LUCAT1 with miR-181a-5p mimic (miR-181a-5p + pc-LUCAT1 group) neutralized the tumor-promoting effects of pc-LUCAT1 (*p* < 0.05, Fig. 4b and c). Meanwhile, miR-181a-5p mimic transfection significantly impaired cell migratory and invasive capacities of MDA-MB-231 cells. In contrast, pc-LUCAT1 transfection induced BC cells migration and invasion. After co-transfection with pc-LUCAT1 and miR-181a-5p mimic, the migratory and invasive capacities of MDA-MB-231 cells were not significantly changed compared with that of cells without transfection (*p* < 0.05, Fig. 4d and e). Colony formation assay and transwell invasive assay were conducted in MDA-MB-231 cells treated with miR-181a-5p mimic, miR-181a-5p mimic + inhibitor, miR-181a-5p mimic + pc-LUCAT1, and miR-181a-5p mimic + pc-LUCAT1 + inhibitor. The results showed that the number of colonies and invasion were gradually increasing. It suggested that miR-181a-5p mediates the effects of LUCAT1 on cell proliferation, migration and invasion in breast cancer (*p* < 0.05, *p* < 0.01, Fig. 4f).

### MiR-181a-5p mediates the tumor-promoting effects of LUCAT1 in vivo

**Fig. 5:**
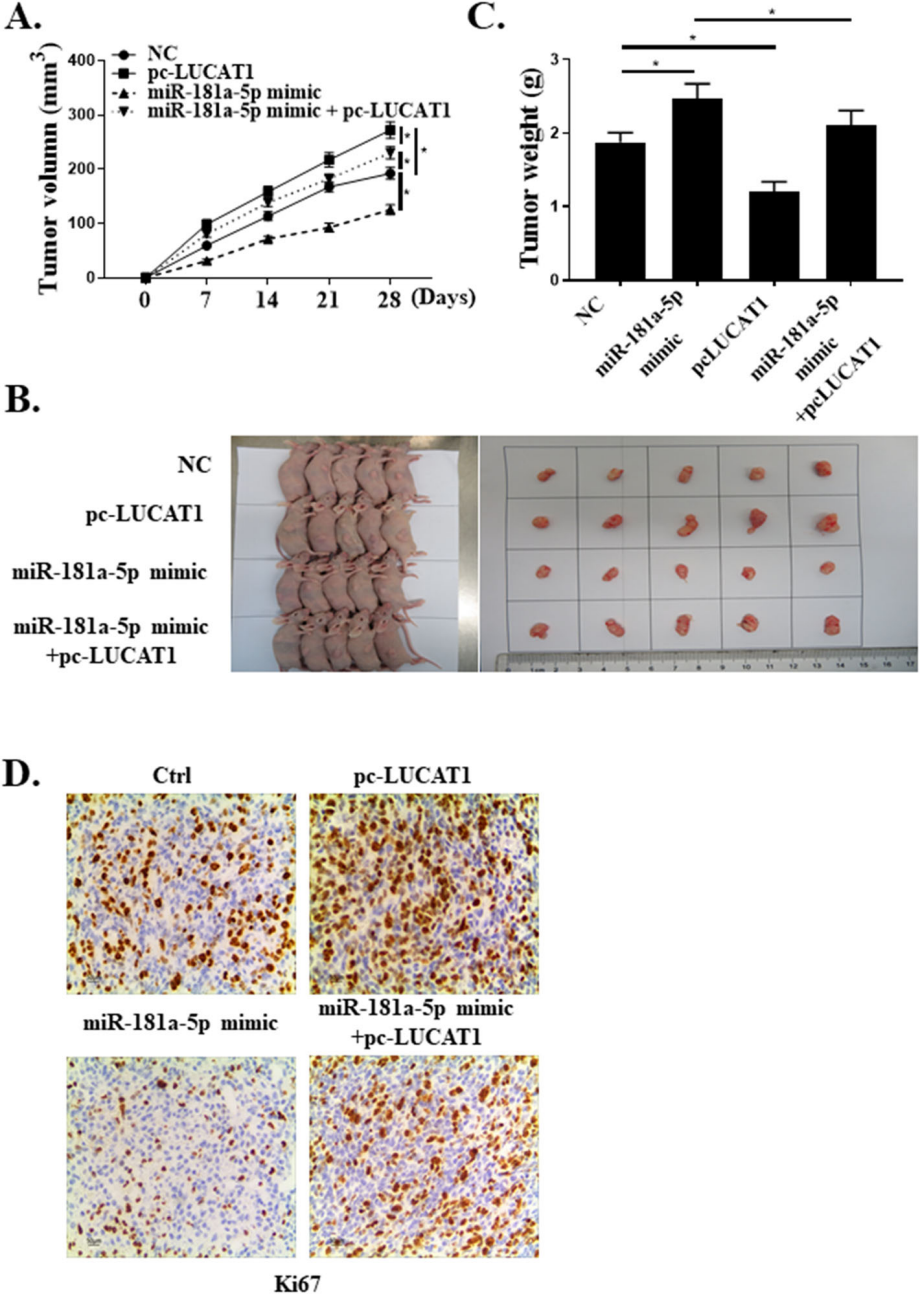
The effects of LUCAT1 on cell proliferation was mediated by miR-181a-5p in vivo. **a** Tumor volumes. **b** The images of tumors. **c** Tumor weights. **d** Proliferation capacity of mouse tumor tissue staining by Ki67. *Compared with Ctrl group, *p < 0.05, n = 5

The role of LUCAT1/miR-181a-5p axis in BC was explored in vivo. It was shown that overexpression of LUCAT1 could promote the growth of mice tumors, whereas overexpression of miR-181a-5p could attenuate tumor growth. The tumor volume of mice in pc-LUCAT1 + miR-181a-5p mimic group was significantly smaller than that in the pc-LUCAT1 group (*P* < 0.05, Fig. 5a-d).

### LUCAT1/miR-181a-5p axis regulates the expression of KLF6 and KLF15

**Fig. 6:**
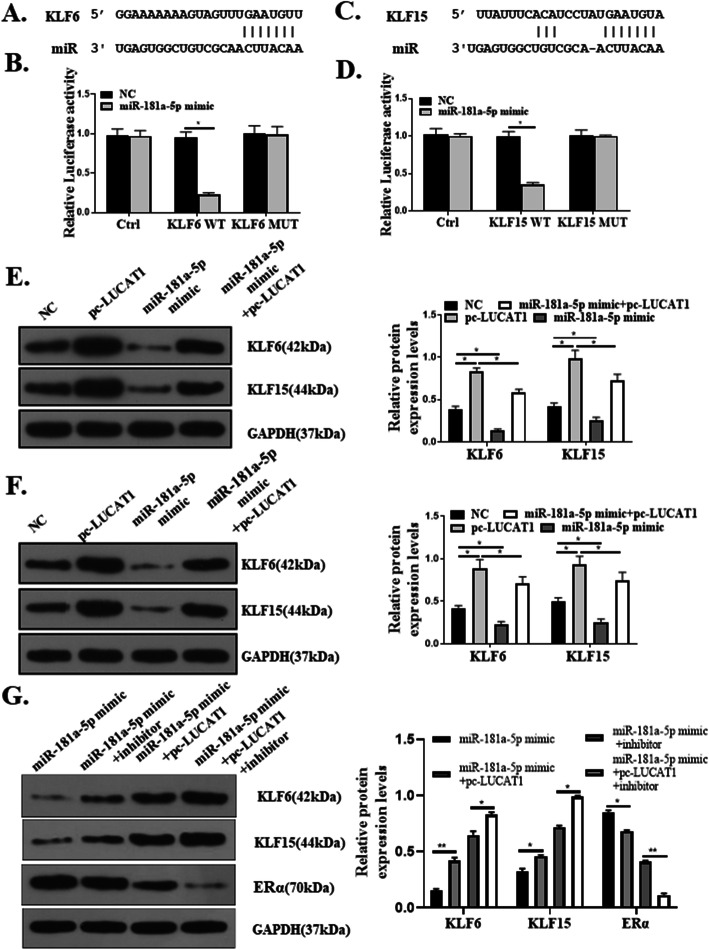
LUCAT1/miR-181a-5p axis regulates the expression of KLF6 and KLF15. **a** and **c** Outputs of the online servers on the binding of miR-181a-5p with the 3’-UTR region of KLF6 and KLF15 mRNAs. **b** and **d** Validation of the binding of miR-181a-5p with KLF6 and KLF15 mRNAs by the 3’-UTR luciferase reporter gene assay. **e** Western blot analysis of KLF6 and KLF15 expression in MDA-MB-231 cells. **f** Western blot analysis of KLF6 and KLF15 expression in mice tumor tissues. **g** Western blot analysis of KLF6, KLF15 and ERα expression in MDA-MB-231 cells. *Compared with Ctrl group, *p < 0.05. **p < 0.01, *n* = 3 or 5

To seek the potential targets of miR-181a-5p, bioinformatic analysis was conducted and the output of miRDB showed that the 3′-UTR region of seven consecutive bases in KLF6 and KLF15 mRNA sequences completely complement with the seed of miR-181a-5p (Fig. 6a and c). The 3′-UTR luciferase reporter gene assay showed that miR-181a-5p pronouncedly inhibited the luciferase activity of pcDNA-KLF6 or pcDNA-KLF15 (*p* < 0.05, Fig. 6b and d). To further validate that KLF6 and KLF15 were targets of miR-181a-5p, western blotting analysis of KLF6 and KLF15 were performed, which showed increased expression levels of KLF6 and KLF15 in cells with pc-LUCAT1 transfection. In contrast, the expression levels of KLF6 and KLF15 were sharply reduced by transfection of miR-181a-5p mimic. The reduction of KLF6 and KLF15 by miR-181a-5p could be reversed by the co-transfection of pc-LUCAT1 and miR-181a-5p mimic together (*p* < 0.01, Fig. 6e). The same results were also observed in mice tumor tissues (*p* < 0.05, Fig. 6f). The expression of KLF6, KLF15 and ERα were detected in in MDA-MB-231 cells treated with miR-181a-5p mimic, miR-181a-5p mimic + inhibitor, miR-181a-5p mimic + pc-LUCAT1, and miR-181a-5p mimic + pc-LUCAT1 + inhibitor. The results showed that the expression levels of KLF6 and KLF15 were increasing, while the expression levels of ERα were decreasing. It suggested that LUCAT1/miR-181a-5p axis regulated the expression of KLF6 and KLF15, and KLFs modulated the expression of ERα (*p* < 0.05, *p* < 0.01, Fig. 6g).

## Discussions

In this study, we explored a novel lncRNA, LUCAT1, as a potential biomarker in BC. Using qRT-PCR analysis, we showed that LUCAT1 was overexpressed in both BC tissues and cells. This finding was consistent with previous reports that LUCAT1 is overexpressed in multiple human cancers [6–9]. Our findings indicated the potential use of LUCAT1 as a diagnostic marker of BC. To further validate the expression of LUCAT1 in predicting BC prognosis, correlation of the expression levels of LUCAT1 with clinical stages is needed to examine whether the expression of LUCAT1 elevated with higher-grade cancer. Instead of analysis on tissue-level LUCAT1, the quantification of serum level LUCAT1 is more convenient and of greater translational potential, which portends further studies.

Further, the value of a LUAT1 as a therapeutic target in BC was demonstrated as the impaired BC cell proliferation by si-LUCAT1 and enhanced BC cell proliferation by pc-LUCAT1. This finding agrees with previous data showing that knockdown of LUCAT1 was able to exert a potent anti-tumor effect [19]. Given the potential of LUCAT1 as a therapeutic target in reversing chemoresistance [6], further studies are also needed to investigate if knockdown of LUCAT1 is a viable strategy to treat chemoresistance BC, which is critical for improving the prognosis of late-stage BC patients.

Contrary to the upregulation of LUCAT1 in BC, miR-181a-5p was shown to be downregulated in BC, and a negative correlation between miR-181a-5p and LUCAT1 was observed. Using bioinformatic analysis and luciferase reporter assay, we confirmed that miR-181a-5p was a target of LUCAT1, which was in agreement with the sponging effects of lncRNAs in regulating expression of miRNAs [20]. This interaction between LUCAT1 and miR-181a-5p translated to the observation that miR-181a-5p mediated the tumor-promoting effects of ectopic overexpression of LUCAT1, as evidenced by the attenuated in vitro BC cell proliferation and in vivo tumor growth after co-transfection of miR-181a-5p mimic and pc-LUCAT1. Previously, LUCAT1 was shown to interact with an array of miRNAs such as miR-375 [19] and miR-200c [6] Therefore, our data cannot rule out that the interaction between LUCAT1 and other miRNAs also contribute to the tumor-promoting effects of LUCAT1.

MiR-181a-5p was previously shown as a therapeutic target in a number of cancers [11, 12]. Here we showed that miR-181a-5p inhibited KLF6 and KLF15. We focused on KLF6 and KLF15 as the interaction between miR-181a-5p and KLF6 has been previously demonstrated in gastric cancer [21], and KLF6 and KLF15 were putative tumor suppressors in BC [15, 16, 22, 23]. Our study provides clear evidence that KLF6 and KLF15 are targets of miR-181a-5p and the regulation of KLF6 and KL15 by miR-181a-5p could be a mechanism of the regulation of miR-181a-5p and LUCAT1 in BC.

## Conclusion

In summary, we demonstrated that the LUCAT1/miR-181a-5p axis constitutes an important regulatory mechanism in BC, whereby LUCAT1 is a tumor inducer and miR-181a-5p is a tumor suppressor. A direct interaction between LUCAT1 and miR-181a-5p exists. The downregulation of KLF6 and KLF15 are also identified as a mechanism of the regulation of miR-181a-5p in BC. LUCAT1/miR-181a-5p is a promising therapeutic target in BC.

## Methods

### Samples

The Ethics Committee of Tongji Hospital approved this study and all experiments were carried out in accordance with the principles of the Declaration of Helsinki. A total of 31 breast cancer patients who underwent tumor resection, but without chemo- or radiotherapy or acute lung/heart/kidney diseases, provided tumor and adjacent normal tissues. All patients were informed of experimental details and signed the informed consent.

### Cell culture and transfection

MCF-10A, MDA-MB-231, MDA-MB-435, MDA-MB-468, SKBR3, MCF-7 and HEK293 cells were acquired from the American Type Culture Collection Company (ATCC; Manassas, VA, USA), and cultured in RPMI-1640 medium (Gibco, Grand Island, NY, USA) supplemented with 10% fetal bovine serum (FBS; Gibco), 100 U/ml penicillin (Gibco), and 100 U/ml streptomycin (Gibco) in a humidified incubator with 5% CO_2_ at 37 °C. MDA-MB-231 cells of 1 × 10^6^ were cultured to 90% confluence in 6-well plates with 2 mL complete medium. Si- LUCAT1, pc-LUCAT1, hsa-miR-181a-5p mimics, hsa-miR-181a-5p inhibitor, and negative control (NC), which were synthesized by Shanghai GenePharma Inc. (Shanghai, China), were transfected into MDA-MB-231 cells by Lipofectamine 3000 in Opti-MEM serum-free medium following the manufacturer’s instructions.

### qRT-PCR

RNAs were extracted using TRIzol reagent (Invitrogen, Carlsbad, CA, USA). RNA samples (200 ng) were reverse transcribed by ReverTra Ace qPCR RT Kit (Toyobo, Japan). THUNDERBIRD SYBR qPCR Mix (Toyobo, Japan) was used for qPCR in a Light Cycler 480 Real-Time PCR system (Roche, Shanghai, China). GAPDH and U6 were used as house-keeping genes. The primer sequences were as follows:

Si-LUCAT1, 5′-CCCAUCAGAAGAUGUCAGAAGAUAA-3′ (sense) and 5′-UUAUCUUCUGACAUCUUCUGAUGGG-3′ (antisense); LUCAT1, 5′-ACCAGCTGTCCCTCAGTGTTCT-3′ (forward) and 5′-AGGCCTTTATCCTCGGGTTGCCT-3′ (reverse); miR-181a-5p, 5′-GCCGAACATTCAACGCTGTCG-3′ (forward) and 5′-GTGCAGGGTCCGAGGT-3′ (reverse); U6, 5′-CTCGCTTCGGCAGCACA-3′ (forward) and 5′-AACGCTTCACGAATTTGCGT-3′ (reverse); GAPDH, 5′-ATGGAAATCCCATCACCATCTT-3′ (forward) and 5′-CGCCCCACTTGATTTTGG-3′ (reverse).

### MTT assay

Cells were seeded in 96-well plates (200 μL, 3 × 10^3^ cells/well) with 10 μL MTT agent (5 mg/mL). After incubation for 4 h, dimethyl sulfoxide (DMSO, 100 μL) was added to dissolve the precipitates, followed by measurement of absorbance at 490 nm with a microplate spectrophotometer.

### Colony formation assay

Cells (1 × 10^3^ cells per well) were seeded in a 6-well plate and incubated for 1 week. After washing with PBS, cells were fixed with 4% formaldehyde for 15 min and stained for 10–30 min with 2.5% Giemsa stain, followed by counting the colonies with a diameter of over 100 μm.

### Transwell assay

Cells were plated in the upper chamber of each insert (3412, Corning, Cambridge, USA) containing the non-coated membrane, with the lower chambers added with medium supplemented with 1% fetal bovine serum (600 μL). After incubating at 37 °C for 24 h, cells in the upper surface of the membrane were removed with a cotton tip, followed by staining of cells on the lower surface with 0.1% crystal violet for 30 min. For the invasion assay, matrigel coated upper chambers (BD Biosciences, San Jose, CA, USA) were used.

### RNA pull-down assay

Flag-MS2bp-MS2bs-based pull-down assay was performed. Briefly, approximately 1 × 10^7^ cells were lysed using RIPA lysis buffer plus 80 U/mL RNasin (Promega, Madison, WI, USA). ANTI-FLAGM-280 Magnetic Beads (Invitrogen) of 50 μl were then added and incubated for 4 h. Lysis buffer was used to wash the beads for 6 times.

### Luciferase reporter assay

For Luciferase reporter assay, 100 ng plasmids and 200 nmol/L miR-181a-5p mimic or their negative control were used to transfect cells (1 × 10^5^ per ml) using Attractene Transfection Reagent (Qiagen). After transfection for 2 d, the promoters of LUCAT1, KLF6 and KLF15 were amplified and inserted into a psiCHECK™-2 vector (Promega), the luciferase activity was evaluated by determining the ratio of firefly to Renilla luciferase activity with a dual-luciferase reporter system (Promega).

### Tumor xenograft in vivo

A total of 30 tumor-bearing nude mice (18–22 g, 6–8-week-old) were purchased from the Animal Center of Tongji Hospital. Animal experiments were conducted following the guidelines for the Protection and Use of Experimental Animals issued by the National Institutes of Health. Animal research is carried out following the scheme approved by the Animal Protection and Utilization Committee of Tongji Hospital. The study was approved by the Ethics Committee of Tongji Hospital. Mice were placed in an animal laboratory without specific pathogens under the following conditions: temperature (23 ± 2 °C), humidity (52.56 ± 2.03%), standard photoperiod (12 h light/dark), free access to food and water. Nude mice were divided into four groups with 5 nude mice in each group. MiR-181a-5p and LUCAT1 overexpression vectors or empty vectors were transfected into MDA-MB-231 cells. We injected 100 μL (5 × 10^6^) cell suspension into the left or right back of each mouse for the tumor formation experiment. The mice in the control group were injected with MDA-MB-231 cells suspension treated with empty vectors. Then use a digital caliper to monitor the size of the tumor once a week. The tumor volume was calculated as follows: volume = (long diameter × short diameter^2^)/2. After 28 d, the mice were euthanized with isoflurane, the mice were sacrificed and the tumor tissues were weighed. Tissue samples were stored in − 80 °C for further analysis.

### Bioinformatics analyses

The prediction of target genes of miR-181a-5p was performed by Starbase (http://starbase.sysu.edu.cn), TargetScan (http://www.targetscan.org/) and miRDB (mirdb.org/miRDB/) bioinformatics analyses, followed by further analysis with the KEGG system. Twenty predicted targets were identified during the first screening. Next, the secondary screening was performed by selection of genes with functional change that induced any changes in LUCAT1, KLF15 and KLF6 in previous studies, which were selected prior to other candidates.

### Western blotting

Total proteins (30 μg) from tumor lysates were separated by 12% SDS-PAGE gel, followed by transferring to PVDF membranes (Millipore, Billerica, MA, USA). The primary antibodies KLF6 (1:300, 67,297–1, Proteintech, China), KLF15 (1:300, ab22851, Abcam, UK),and GAPDH (1:800, ab8245, Abcam, UK) were used to incubate with the membrane at 4 °C overnight. After incubation for 1 h with the HRP-conjugated secondary antibody, an ECL Kit (Millipore) and a UV Transilluminator (Bio-Rad) were used to acquire and analyze the protein band intensities.

### STR genotype detection

DNA was extracted by Axygen genome extraction kit (Corning) and amplified by 21-STR amplification scheme. STR point and sex gene Amelogenin were detected on the ABI3730XL genetic analyzer.

### Statistical analyses

GraphPad Prism 6.0 (GraphPad Software) was used for data analysis. Data were expressed as mean ± standard deviation (SD). Differences between individual groups were analyzed by Student’s t-test. The criterion of statistical significance was *p* < 0.05.

## Data Availability

The datasets used and/or analyzed during the current study are available from the corresponding author on reasonable request.

## Abbreviations

siRNA: small interfering RNA
lncRNAs: long non-coding RNAs
BC: Breast cancer
miRNAs: microRNAs
